# Erosion of the Atheroma: Wicked T Cells at the Culprit Site

**DOI:** 10.1007/s11883-024-01247-x

**Published:** 2024-11-16

**Authors:** Shiying Lin, Yinda Yu, Leif Å Söderström, Anton Gisterå

**Affiliations:** 1 Department of Medicine Solna, Center for Molecular Medicine, Karolinska University Hospital, Karolinska Institutet, Stockholm, Sweden; 2 Karolinska University Hospital, Visionsgatan 4, Solna, Stockholm, SE-17164 Sweden

**Keywords:** Atherosclerosis, T-lymphocytes, Plaque erosion, Extracellular matrix, Cardiovascular disease

## Abstract

**Purpose of Review:**

There is a growing recognition of plaque erosion as a cause of acute coronary syndrome. This review aims to examine the potential involvement of T cells in this process.

**Recent Findings:**

Immune-vascular interactions have been identified in the development of plaque erosions. Up to one-third of eroded plaques show evidence of active immune infiltration, with the presence of T cells. We propose that microerosions may frequently occur in association with the infiltration of T cells and macrophages in early atherosclerotic lesions. Healing of erosions could trigger the deposition of excessive extracellular matrix. The pro-inflammatory and cytotoxic actions of T cells, along with reduced endothelial integrity and other mechanisms, may subsequently give rise to clinical symptoms.

**Summary:**

To gain a better understanding of the role of T cells in plaque erosion, it is crucial to develop improved models for conducting controlled experiments and to study atherosclerosis in younger individuals.

## Introduction

Atherosclerotic plaque erosion is increasingly recognized, with recent studies showing that it occurs in up to 40% of acute coronary syndromes [1]. This poses a significant medical problem, considering that approximately 220 million people under the age of 60 have experienced a myocardial infarction worldwide [2]. However, the specific mechanisms that cause plaque erosion are still poorly understood, and there is a growing number of observational and mechanistic studies addressing this knowledge gap. As the research field continues to mature, we have taken the opportunity to examine the most interesting findings related to the debated topic of inflammation in plaque erosion (Table 1). Observational studies support the notion that activation of inflammatory pathways is involved in the pathophysiology leading to plaque erosion. However, the role of T cells in these processes has not been previously reviewed. A couple of classical references shed light on their presence in the culprit lesions [3, 4], and new studies are now focusing on identifying mechanistic roles for T cells that align with the concept of plaque erosion [5, 6].

**Table 1:** Recent atherosclerotic plaque erosion reports

Erosion reports 2019-2024	Type of study	Summarized finding
Fang et al. [37]	Optical coherence tomography of 1442 ST-elevation myocardial infarction cases.	Patients under the age of 50 more frequently had plaque erosion and less rupture-prone plaques.
Yamamoto et al. [52]	Optical coherence tomography of 18 plaque erosion cases with calculations of endothelial shear stress.	The thrombus was found in areas of high shear stress and extended into regions of low shear stress. It was also located near steep plaque shoulders.
Yamamoto et al. [1]	Optical coherence tomography of 1241 acute coronary syndrome cases.	Plaque erosion was found to be associated with non-ST elevation myocardial infarction, age less than 68 years, anterior ischemia, the absence of diabetes mellitus, hemoglobin levels greater than 150 g/L, and normal kidney function.
Kurihara et al. [22]	Optical coherence tomography of 1113 acute coronary syndrome cases.	A higher proportion of plaque ruptures occur in winter, while a higher proportion of plaque erosions occur in summer.
Leistner et al. [6]	Optical coherence tomography of 170 plaque erosion cases with immunophenotyping in blood	Enrichment of CD4 + and CD8 + T cells was observed in coronary blood samples as compared to peripheral blood. Additionally, CD8 + T cells were found to be abundant in aspirated thrombi.
Lupieri et al. [5]	Endovascular injury to C57BL6/J and phosphoinositide 3-kinase γ kinase-dead mice	Phosphoinositide 3-kinase γ kinase-dependent T-cell response leads to the production of C-X-C motif chemokine ligand 10 by smooth muscle cells, which in turn inhibits endothelial healing.
Araki et al. [23]	Optical coherence tomography of 648 ST-elevation myocardial infarction cases.	No circadian variation was detected in cases of plaque erosion, while the incidence of plaque rupture peaked at 9 AM.
Gao et al. [76]	Ultrasound-activated nanoparticles targeting macrophages and activated platelets in *Apoe*^−/−^ mice.	Apoptosis of macrophages and disaggregation of activated platelets locally provide potential strategies to target plaque rupture and erosion, respectively.
Kato et al. [39]	Carotid ultrasound and coronary optical coherence tomography of 115 acute coronary syndrome cases.	Carotid arteries had less intima-media thickness and heterogenous plaques (representing rupture-prone characteristics) in plaque erosion cases.
Molinaro et al. [77]	Type IV collagen-targeted nanoparticles delivered protein arginine deiminase-4 inhibitor to denuded endothelium in *Apoe*^−/−^ mice.	The treatment reduced the presence of neutrophil extracellular traps at the sites of intimal injury, leading to an improvement in endothelial healing.
Nakajima et al. [75]	One-year follow-up with optical coherence tomography of 49 plaque erosion cases treated with or without glycoprotein IIb/IIIa inhibitor during the procedure.	The use of glycoprotein IIb/IIIa inhibitors was associated with a lower rate of atherosclerosis progression and reduced formation of layered plaques.
Sekimoto et al. [9]	Optical coherence tomography of 62 plaque erosion cases.	A subclassification dividing erosion cases into fibrous (31%) and lipid-rich (69%) culprit plaques.
Wang et al. [34]	Optical coherence tomography of 274 ST-elevation myocardial infarction cases and 2.2 years follow-up for recurrent cardiovascular events.	Both the morphology of the culprit plaque and the residual atherosclerosis burden improved the predictability of recurrent cardiovascular events.
Yin et al. [8]	Optical coherence tomography of 117 plaque erosion cases.	Layered plaques, which indicate a healed phenotype, were identified in 48% of cases and were found to be associated with a higher prevalence of macrophage infiltration.
Gerhardt et al. [30]	Optical coherence tomography of 398 acute coronary syndrome cases and 2 years follow-up for recurrent cardiovascular events.	Plaque erosion cases had lower rates of recurrent events and lower plasma interleukin-6 levels, while plasma C-reactive protein and interleukin-1β levels were similar to those in plaque rupture cases.
Kawai et al. [70]	Detection of X-chromosome inactivation patterns in smooth muscle cells in coronary sections from 3 plaque erosion autopsy cases.	The clonality of smooth muscle cells was calculated to be 27% in cases of plaque erosion, indicating that most of the smooth muscle cells have a polyclonal origin.
Meteva et al. [68]	Optical coherence tomography of 32 plaque erosion cases and matched plaque rupture cases with an aspiration of thrombi and collection of local and peripheral blood.	Cases of plaque erosion showed an increase in toll-like receptor 2 expression on neutrophils, a higher concentration of matrix metalloproteinase 9 and hyaluronan locally, and elevated expression of hyaluronidase 2 in aspirated thrombi. In culture, neutrophils from plaque erosion cases exacerbated endothelial cell death.
Satta et al. [54]	Coronary artery endothelial cells cultured under elevated shear stress and exposure to cigarette smoke extract.	Elevated flow, cigarette smoke extract, and inflammation can trigger the detachment of endothelial cells in culture, replicating features of plaque erosion in patients.
Seegers et al. [35]	Optical coherence tomography of 382 female acute coronary syndrome cases.	Plaque erosions were predominant in females under 60 but decreased with age. The presence of macrophages, however, was not influenced by age.
Weng et al. [38]	Carotid, femoral, and popliteal ultrasound and coronary optical coherence tomography of 297 acute coronary syndrome cases	Plaque erosion cases had fewer and smaller peripheral plaques, which were also less heterogeneous (representing rupture-prone characteristics).
Zeng et al. [43]	Optical coherence tomography of 190 myocardial infarction cases with nonobstructive coronary arteries.	Atherosclerotic plaques were present in 52% of the cases, and plaque erosion was found in 65% of those cases. The majority of plaques showed the presence of macrophages.
Cornelissen et al. [40]	Histopathological and polygenic risk score analysis of 945 sudden cardiac death autopsy cases.	Coronary artery disease risk loci were found to be associated with overall plaque burden and plaque rupture but did not show any association with plaque erosion.
Li et al. [19]	Optical coherence tomography of 312 ST-elevation myocardial infarction cases with aspiration of thrombi.	Plasma and thrombus levels of aldehyde dehydrogenase 4A1 were higher in cases of plaque erosion.
Molinari et al. [73]	Electrical injury-induced carotid erosions in bone marrow chimeric *Lyz2*-Cre *Jak2*^V617F^*Ldlr*^−/−^ mice.	The clonal hematopoiesis mutation *Jak2*^V617F^ was found to increase neutrophil extracellular traps and cause impairment in endothelial integrity in mice.

Although eroded plaques have fewer immune cells compared to ruptured plaques, it is still important to consider their role in the development of the disease, especially because T cells are abundantly present in the early stages of atherosclerosis. The interaction between immune cells and vascular cells provides a promising area for further research [7]. In certain cases of plaque erosion, there is a significant presence of T cells, and recent studies have suggested that these cells might directly contribute to the manifestation of the disease.

Observational studies of human plaques still have much to teach us. In these cases, larger studies with greater statistical power are not necessarily beneficial. Smaller studies, on the other hand, provide the opportunity for more detailed analysis and better control of quality. This is particularly crucial when studying adaptive immunity in atherosclerosis, as it involves both pro-inflammatory and anti-inflammatory properties, and the overall balance determines the outcome. The key to understanding lies in the details, and recent advancements in plaque erosion research have aided in defining underlying subclasses of plaques related to this manifestation [8, 9]. The question is whether this simply represents a range of different morphologies of atherosclerotic plaques, or if subclassification has clinical significance and can lead to new treatment approaches with better outcomes. Our review emphasizes the role of T cells in the development of atherosclerosis. To further this research and validate the proposed concepts regarding plaque erosion, we need improved research tools that can assess the function of different subsets of T cells throughout the progression of the disease.

## Progression of Atherosclerosis toward Plaque Rupture or Erosion

Although atherosclerosis is considered a single disease entity, the morphology of plaques can vary significantly. This has been studied in great detail using histological techniques [10]. The development of smoldering inflammation in response to retained lipoproteins in the intimal layer over decades likely contributes to the diversity [11]. With age, the atherosclerotic plaque becomes more complex. According to current beliefs, fatty streaks develop into fibro-fatty lesions, which then progress into advanced plaques [10]. Ultimately, there is a risk of plaque rupture or erosion in a coronary artery, which can result in thrombosis and acute coronary syndromes [12].

Plaque ruptures are still the primary cause of coronary thrombosis events [13]. The cytokines produced by macrophages and T cells play a critical role in driving the production of proteases and hindering the actions of proteolytic inhibitors in the plaques. This leads to the formation of a large necrotic core and a thin fibrous cap, which are key features of a plaque prone to rupture [14]. Modified lipoproteins, cholesterol crystals, or other antigenic molecules derived from modified proteins, lipids, or microbes, are drivers of this process [15]. Autoimmune reactions against self-peptides are also evidently part of these processes [16, 17]. Since up to 40% of culprit lesions have intact fibrous caps, we have to consider other mechanisms than plaque rupture as well. The driving forces and pathological mechanisms behind this are likely different from the ones causing plaque rupture. For example, aldehyde dehydrogenase 4A1 is a mitochondrial autoantigen expressed in atherosclerotic plaques [18]. It has been associated with the progression of atherosclerosis and is suggested to be released from its secluded mitochondrial niche during tissue damage. Elevated levels have been detected in blood from plaque erosion patients and in aspirated thrombi [19]. This indicates that plaque erosions might help induce maladaptive immune responses in atherosclerosis, or vice versa.

The development of atherosclerosis is often described as a linear process [20]. However, in reality, it is likely a more dynamic process. Plaque progression and regression can occur interchangeably in the vasculature. Intermittent exposure to risk factors and proinflammatory stimuli can predispose to rapid evolution phases [11]. This is followed by stable phases, with the resolution of inflammation, stabilization processes, and even regression of plaques at times. Clinical studies using intravascular imaging support this idea, demonstrating that coronary plaques can switch between vulnerable and non-vulnerable phenotypes [21]. Further support for this notion is provided by the observation that seasonal variations occur, with plaque erosion being more common in the summer [22]. Unlike plaque ruptures, which occur more frequently in the morning and display circadian variation, plaque erosion does not exhibit any circadian variation [23]. Taken together, this suggests that the transition between erosion-prone and rupture-prone plaques is a slower process compared to the transitions between stable and vulnerable plaques.

## Inflammatory Involvement in Plaque Erosion

Plaque erosions are typically defined as culprit lesions with intact fibrous caps [24]. The underlying intima generally contains abundant connective tissue and smooth muscle cells. Most erosions occur over areas of intimal thickening, with small or no lipid cores. Plaques that are rich in matrix but poor in lipids usually do not exhibit prominent macrophage populations. Previous reviews have suggested that multiple processes contribute to the predisposition of these plaques to superficial erosion [25–27]. These processes include flow disturbance, breakdown of the basement membrane, death of endothelial cells, and their detachment potentiated by innate immune activation and endothelial-mesenchymal transition.

An investigation conducted in the 1990s examined 50 cases of sudden coronary death [4]. It was discovered that 22 of these cases involved coronary thrombi without plaque rupture. Notably, all of these cases showed signs of superficial erosion in a plaque that was rich in proteoglycan. The patients with plaque erosions were younger and more often female compared to the 28 cases of plaque rupture. Additionally, the eroded plaques were less calcified and had a lower stenosis grade. T-cell infiltration in the fibrous cap was found in 75% of plaque rupture cases and 32% of plaque erosion cases. The antigen-presenting molecule, human leukocyte antigen DR isotype, was present in 89% of ruptured plaques and 36% of eroded plaques. These findings suggest that fewer immune cells are observed in plaque erosions compared to plaque rupture, thus indicating that inflammation may play a lesser role in the development of erosion-related disease manifestations. Clinical studies have also shown lower systemic levels of C-reactive protein in patients with plaque erosion compared to those with plaque rupture [1, 28]. However, a smaller study found equal concentrations of C-reactive protein comparing plaque rupture and erosion [29], indicating that vascular inflammation can be significant in individuals with fatal plaque erosions. Another cohort study also found C-reactive protein and interleukin-1β to be similar between plaque rupture and erosion in living subjects [30]. However, it is important to note that the acute inflammatory response does not provide a complete understanding of the processes leading to disease manifestations. Additionally, infiltrates of activated mast cells have been detected at the site of coronary plaque erosions, indicating active inflammatory processes in the culprit lesions [31]. Taken together, histological studies of autopsy material have shown that up to a third of plaque erosion cases exhibit active immune infiltration, with the presence of T cells whose role remains largely unknown.

## Clinical Presentation of Plaque Erosions

Plaque erosions have a distinct clinical presentation compared to plaque rupture. Patients with plaque erosion have fewer conventional cardiovascular risk factors, such as diabetes, dyslipidemia, chronic kidney disease, and hypertension [25]. This suggests that managing traditional risk factors alone may not be sufficient to prevent plaque erosions. In fact, there is evidence that endothelial erosions are increasing [32]. Advanced diagnostic tools and modern pharmaceutical treatments, such as statins, that alter plaque composition may contribute to this change [33]. Similar factors may also explain the decrease in ST-segment elevation myocardial infarction and the simultaneous increase in non-ST-segment elevation myocardial infarction [32]. However, it is important to note that recurrent events are more common after plaque rupture than after plaque erosion, highlighting the continued significance of plaque rupture in medical research [34].

Plaque erosion tends to affect younger populations, particularly females and often manifests as a non-ST-segment elevation myocardial infarction [35]. Smoking, especially in premenopausal females, has been linked to plaque erosions [24, 36]. Specifically, when looking at ST-segment elevation myocardial infarction, plaque erosions account for a higher proportion of cases in young patients (≤ 50 years) compared to older patients (32% vs. 21%) [37]. This study also reveals that young patients are less likely to have lipid-rich plaques with thin caps, calcification, and cholesterol crystals, which aligns with the progression of atherosclerosis where more complex lesions develop with age. It suggests that plaque erosions occur earlier in the atherosclerosis process. Apart from inflammation, smoking is the only modifiable risk factor that stands out. Therefore, it is crucial to focus on smoking cessation and prevention strategies targeting inflammation in younger populations.

## Connecting Coronary Plaque Erosion with Other Vascular Beds

We propose that plaque erosion could be considered an earlier manifestation than plaque rupture in many cases. This is illustrated by the fact that patients with coronary plaque ruptures have more frequent plaques in other locations compared to patients with coronary plaque erosions [38]. Patients with plaque erosion also have smaller carotid plaques compared to those with calcified plaques [39]. However, this study did not find a significant difference in carotid plaque size when comparing coronary plaque erosion and rupture.

The similarity in the size of plaques between vascular beds in cases of plaque erosion suggests that there are shared factors contributing to plaque development. Therefore, it is important to not only consider local conditions but also take into account systemic processes like inflammation and genetic factors. In this context, it is worth noting that polygenic risk scores are closely associated with the overall burden and rupture of atherosclerotic plaques [40]. However, this polygenic risk score showed no consistent associations with plaque erosion. It could imply different causes for plaque ruptures and erosions and it suggests that erosion may be driven by non-genetic factors or by genetic risk loci other than those included in this particular polygenic risk score calculation. Arguably, there are genetic factors for plaque erosions, as females with a family history of coronary artery disease have more plaque erosion [35]. However, a separate genome-wide association study exclusively focused on patients with plaque erosion has not been performed so far. Moreover, these large-scale genetic studies do not provide information regarding the potential role of specific T cells in plaque erosion etiology, and the control subjects are not screened for subclinical atherosclerosis.

## Advancements in Imaging of Plaque Erosion

Currently, the imaging techniques used mostly identify the physical features of plaques, but they are not capable of detecting the molecular characteristics that are known to be important drivers of future risk [41]. Coronary angiography is the standard method for assessing coronary stenosis; however, it has limitations in evaluating coronary plaque morphology beyond size [42]. Computer tomography angiography, which primarily focuses on measuring plaque calcification, may also overlook erosion-prone plaques due to the absence of calcification in these plaques. Instead, the most frequently used method for studying plaque erosion is optical coherence tomography. This light-based intravascular imaging technique can provide information on coronary plaque morphology with a resolution of 10–20 μm. It can characterize fibrous, fibrocalcific, and lipid-rich plaques. Additionally, it can help differentiate the underlying causes of coronary thrombosis by visualizing the disrupted or intact fibrous cap of the culprit lesion [13]. The imaging depth of optical coherence tomography is < 2 mm, which is sufficient for many coronary plaques but inadequate for larger ones. This technique has demonstrated that atherosclerotic lesions are often missed by conventional coronary angiography [43], and in cases of myocardial infarctions with nonobstructive coronary arteries, plaque erosions with intact fibrous caps have been identified as a major cause.

Optical coherence tomography has made it possible to identify rupture-prone plaques by determining cap thickness and the size of the underlying lipid core. It can also provide information on fibroatheroma, macrophages, vasa vasorum, and cholesterol crystal contents [41]. This is currently an area of intensive research for predicting cardiovascular risk and identifying features of erosion-prone plaques. The use of optical coherence tomography for routine diagnostic purposes is, however, impractical due to the need for a specific intravascular imaging probe and the required contrast agents. Biomarkers have been suggested as a means to customize the utilization of optical coherence tomography and reduce the risks associated with this invasive technique [25]. Nevertheless, there is still potential for improvement in terms of resolution and imaging depth. For instance, optical coherence tomography, due to its limited resolution, is unable to directly examine the endothelium, which plays a crucial role in plaque erosion.

Some discrepancies exist between autopsy studies and more recent optical coherence tomography studies. These differences can be attributed to technical factors in histology, limitations of the imaging modality, and underlying patient characteristics. A multicenter optical coherence tomography study investigating 1241 acute coronary syndrome patients did not find sexual dimorphisms or an association between plaque erosion and smoking [1]. In this study, a multivariable logistic regression model identified five independent parameters associated with plaque erosion: age < 68 years, anterior ischemia, absence of diabetes mellitus, hemoglobin > 150 g/L, and normal kidney function. If all five parameters were present in a patient with non-ST-segment elevation myocardial infarction, the likelihood of plaque erosion was 73%. This finding could be valuable in guiding clinical decision-making, as it could be beneficial to approach patient management differently in erosion cases. From a risk management perspective, however, these factors are not useful.

## Imaging of Inflammation Enhances Understanding of Plaque Erosion

Histological properties are still considered the standard for comprehending plaque composition and categorizing plaques. Deep learning models are currently being developed to improve the interpretation of plaque composition. For instance, tools for intravascular imaging techniques are being created to automatically categorize atherosclerotic plaques [44, 45]. This will play a crucial role in translating more advanced histological findings into clinical practice.

Since no imaging modality could detect T cells in plaques, characterizing inflammation in clinical studies remains indirect and incomplete in this regard. However, macrophage patterns can be investigated using optical coherence tomography, which correlates with immunohistochemical identification (*r* = 0.84) [46]. These macrophage patterns are present in 55% of plaque erosions and 80% of plaque ruptures [1].

A recent study suggests that optical coherence tomography can classify plaque erosion into two subgroups [9]. These subgroups are classified as classical fibrous erosions and lipid-rich erosions. The latter subgroup exhibits signs of containing a significant amount of macrophages. Culprit lesions resulting from plaque erosion can also be categorized based on evidence of previous events; recent research using optical coherence tomography identified healed cap patterns in approximately half of the culprit lesions [8]. These patterns indicate prior immune cell infiltration [47]. T cells may have been present, but this could not be determined using optical coherence tomography.

There is a need to study the various stages of subclinical atherosclerosis in order to diagnose the condition and monitor the impact of primary prevention strategies [48]. Near-infrared imaging can identify signs of inflammation by using fluorescent contrast agents that target different inflammatory cascades [41]. However, improved tools are required to examine the presence of T cells in specific locations as the disease progresses. In the future, we hope to be able to detect specific T cell subsets and molecular characteristics of the plaques using positron emission tomography or fluorescent probes.

## Classical Predilection Sites for Plaque Erosions

The most common site for plaque erosion identified so far is the proximal left anterior descending coronary artery at bifurcation points [1, 49]. This is a classical location for atheromatous development, suggesting that flow disturbances are a significant contributor to plaque buildup. Local shear stress affects the apoptosis of luminal endothelial cells and may be a major factor in plaque erosion. Analysis of endothelial cell apoptosis in plaques has shown the occurrence of apoptosis in the downstream parts of the plaques, where low flow and low shear stress are present [50]. However, erosions can occur both proximal and distal to the minimal lumen area of the plaque [51].

**Fig. 1:**
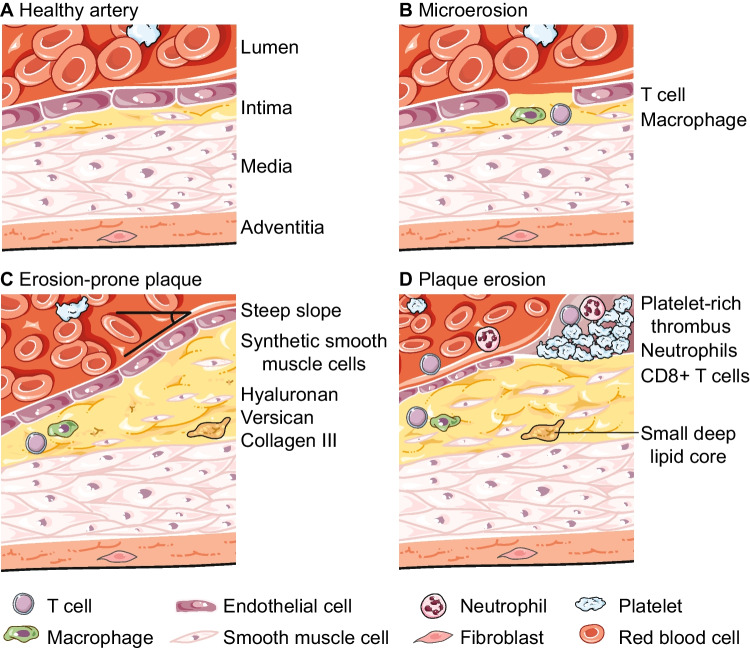
T cells and the progression of atherosclerosis towards plaque erosion. (**A**) In a normal artery, smooth muscle cells are sparsely distributed in the tunica intima, tightly distributed in the tunica media, and loose connective tissue is found in the tunica adventitia. (**B**) In a microerosion, a few endothelial cells are lost over the infiltration site of a T cell and macrophage. (**C**) Factors associated with plaque erosions include a steep slope of the plaque, creating high shear stress, and an extracellular matrix enriched in type III collagen, hyaluronan, and versican. (**D**) An eroded plaque is characterized by a platelet-rich thrombus and a small lipid core, which is not in contact with the thrombus. The erosion site contains neutrophils and CD8 + T cells. The illustration was generated using Servier medical art, licensed under a creative commons attribution 3.0 unported license

Recent studies combining optical coherence tomography with computational biomechanical models have revealed that plaque erosion occurs around atheromatous plaque throats with specific stress patterns [49]. High shear stress may play a crucial role in initiating thrombotic processes, and both shear stress and plaque geometry may determine the location and extent of thrombus formation [52]. The slope of the plaque topography may also contribute to plaque destabilization. The steepness of the slope has been suggested as a possible trigger for the development of plaque erosion (Fig. 1C). However, the factors that determine different slopes are largely unknown, and this concept is not valid for microerosions that might occur in nascent lesions. The sites located proximal and distal to the minimal lumen area of eroded plaques typically experience low shear stress and turbulent flow. These conditions contribute to the development of atherosclerosis and promote the infiltration of T-cells into the vessel wall. It would be beneficial to use the rapidly developing three-dimensional histology techniques to map the distribution of T-cells within eroded plaques. This approach would provide valuable insight into plaque structure, cell interactions, and compartmentalization.

## Endothelial Interactions with Plaque T Cells

Although endothelial cells are not part of the actual atherosclerotic lesion, they play an intricate role in the disease process and come into direct contact with the plaque. Endothelial dysfunction is a critical factor in the development and progression of atherosclerosis [53]. The integrity of the endothelium is also considered the foundation of the plaque erosion concept. For instance, cigarette smoke has been found to trigger endothelial detachment and might therefore directly precipitate erosions [54]. The glycocalyx, which overlays the endothelium, serves as the first line of defense in maintaining endothelial integrity. The role of the glycocalyx in regulating endothelial communication is not well-studied, but it may mediate various vasculoprotective functions of endothelial cells [55]. The breakdown of the glycocalyx due to flow-mediated patterns can lead to increased infiltration of lipoproteins and immune cells into the vascular wall. Given the potential therapeutic implications, we find the study of glycocalyx restoration and extracellular matrix generation in relation to endothelial erosion intriguing.

Activated T cells are a significant cell population within most atherosclerotic plaques [56]. These T cells are antigen-experienced memory cells that show evidence of oligoclonal expansion. CD8 + cytotoxic T cells are essential in combating infected or damaged cells. When their specific T-cell receptors identify an antigen presented on major histocompatibility complex class I, they can release cytotoxic or proinflammatory molecules such as interferon-γ [57]. When cytotoxins enter the targeted cell, apoptosis ensues through their serine protease activity. Cytotoxic T cells can also induce programmed cell death by expressing the Fas ligand, which binds to CD95 on the target cell.

A recent study has highlighted the involvement of CD8 + T cells in the pathogenesis of plaque erosion [6]. The study investigated culprit lesions of 170 consecutive acute coronary syndrome patients using optical coherence tomography. Within this study cohort, 25% of the lesions had intact fibrous caps. Simultaneous analysis of coronary and peripheral blood samples included immunophenotyping by flow cytometry. Both CD4 + and CD8 + T cells were locally present in connection with plaque erosions, with higher frequencies observed in the local blood compared to the peripheral blood. Additionally, more CD8 + T cells were found in thrombi aspirations from endothelial erosion plaques (Fig. 1D). Furthermore, the effector molecules granzyme A, perforin, and granulysin were enriched in the local blood associated with eroded plaques. The authors go on to demonstrate that disturbed flow contributes to T-cell adhesion to endothelium and that T-cell effector molecules can induce endothelial cell death. This is proposed as a key pathophysiological mechanism in endothelial erosion. However, it needs to be validated in controlled experiments where the plaque erosion process can be followed. Histopathology studies of eroded plaques commonly show evidence of thrombus remodeling, indicating that the underlying erosions may have occurred up to a week before the clinical event [27]. This indicates that the CD8 + T cells might be responding to local tissue damage rather than causing the acute event in the first place.

In mice, the overall impact of CD8 + T cells on atherogenesis has not been conclusive [7]. The pro-atherosclerotic impact of interferon-γ-secreting CD4 + T cells has been more evident. Throughout the disease, an anti-inflammatory regulatory T-cell population is also present [58]. Their main effector cytokines are interleukin-10 and transforming growth factor-β. Interestingly, transforming growth factor-β contributes to endothelial inflammation [59]. While transforming growth factor-β has beneficial anti-inflammatory effects on most cells in the actual plaques, it may contribute to a detrimental role in plaque erosion. Recently, the safety of low-dose interleukin-2 administration in patients with ischemic heart disease was evaluated in a clinical trial. The tested doses of interleukin-2 successfully increased regulatory T cells with no significant adverse events [60]. However, further research is required to confirm the safety and evaluate the effectiveness of low-dose interleukin-2 as an anti-inflammatory treatment for individuals with ischemic heart disease. Prior to this, it would be interesting to test whether this therapy has different effects on plaque rupture and erosion in a model system.

## T Cells Direct the Evolution of Plaques into Erosion

Studying the evolution of plaques in atherosclerosis has been challenging, with most of our knowledge based on snapshots of histopathology data [10]. However, single-cell sequencing has started to expand our understanding of these processes and highlights interactions between T cells and smooth muscle cells to be important for disease progression [56]. The presence of activated T cells at predilection sites in the arterial intima during the initial stages of the disease has been observed in an autopsy study [61]. CD4 + T cells were found to be predominant in early lesions, while CD8 + T cells showed a slight predominance in advanced lesions. Interestingly, a Russian pathology report suggests that T-cell infiltration may occur in the early stages of atherosclerosis at sites where only a single or a few endothelial cells are damaged [62]. Both T cells and macrophages have been detected at these microerosion sites in the coronary arteries (Fig. 1B). Although microerosions are not expected to cause clinical symptoms, they indicate that erosion is not exclusively a final maturation stage for atherosclerotic plaques. If microerosions occur during the early development of the plaques, it suggests an ongoing process that requires recurrent healing. Biologically, this healing phase may result in the deposition of excessive extracellular matrix, leading to the formation of a plaque with abundant type III collagen and hyaluronan. According to this concept, T cells might play a role in the progression of plaques into erosions but this needs to be verified in further studies.

## Communication Between T Cells and Extracellular Matrix in Plaques

Normal wound healing progresses through four major, interconnected phases: hemostasis, inflammation, proliferation, and remodeling. The proliferation phase involves the formation of new provisional extracellular matrix components, such as collagen, glycosaminoglycans, and hyaluronan. Type III collagen is synthesized in the early stages of wound healing and is subsequently replaced by type I collagen [63]. Therefore, it is interesting to note that the composition of the extracellular matrix differs significantly between plaque erosions and ruptures. Ruptured plaques mainly contain type I collagen, biglycan, and decorin, while erosion plaques have more type III collagen, versican, and hyaluronan [64]. Given the analogy to wound healing, this suggests that plaque erosions occur earlier in the process than plaque rupture, which aligns with the observation that eroded plaques are smaller and occur in younger patients. The viscous hyaluronan- and versican-rich matrix in plaque erosion could restrict T cell migration [65], thereby explaining the relative absence of T cells within eroded plaques.

Versican is a large extracellular chondroitin sulfate proteoglycan, consisting of an N-terminal domain that binds hyaluronan and a central domain decorated with glycosaminoglycans. In contrast, hyaluronan is a large polysaccharide with non-sulfated glycosaminoglycans. CD44 is a receptor for hyaluronan and is expressed on several cell types in the plaque [66]. Fragmentation of hyaluronan by hyaluronidases during tissue injury triggers inflammatory responses. These enzymes are produced by immune cells to facilitate their migration through hyaluronan-rich matrices. Hyaluronan degradation products transmit inflammatory signals through toll-like receptors, which could directly contribute to endothelial cell injury and precipitate plaque erosion [67, 68]. Elevated levels of Hyaluronidase 2 mRNA and CD44 splicing variants can be measured in peripheral blood mononuclear cells of patients with plaque erosion, indicating that these are potential biomarkers [69].

Further investigation is warranted to understand the interaction of T cells with different compositions of the extracellular matrix in atherosclerosis. CD44 is expressed on effector-memory T cells, and hyaluronan may modulate the immune response by promoting the polarization of interferon-γ-secreting T cells [66]. In the plaques, T cells and their secreted interferon-γ inhibit collagen synthesis and smooth muscle proliferation [7]. On the other hand, high molecular weight hyaluronan interacts with CD44 on regulatory T cells, promoting their activation and function, in part by increasing interleukin-10 secretion [65]. This indicates that hyaluronan in plaques plays a role in balancing adaptive immune responses.

## Interactions between T-Cells and Smooth Muscle Cells

Smooth muscle cells are the primary producers of collagens in atherosclerotic lesions. Throughout the progression of atherosclerosis, local expansions of smooth muscle cell clones have been observed, although most smooth muscle cells appear to have a polyclonal origin. In a recent study, an intricate lineage tracing approach was used to demonstrate that 27% of smooth muscle cells in eroded plaques have a clonal origin with spatial distribution in specific regions [70]. Specific RNA imaging probes were applied to sections of coronary arteries from heterozygous females with the deletion of the X chromosome gene BGN, allowing for the visualization of smooth muscle cells with similar X-chromosome inactivation patterns. The clonal frequency of smooth muscle cells in ruptured plaques was slightly higher at 39%. However, how different subsets of T-cells affect the clonality and distribution of smooth muscle cells in plaques is yet to be determined.

Medial smooth muscle cells exhibit a quiescent and contractile phenotype. In atherosclerosis, smooth muscle cells undergo a phenotype switch upon migration into the intima. These cells lose the expression of contractile genes and acquire synthetic properties that contribute to the remodeling of the vascular extracellular matrix. It is worth noting that T-cell-derived interferon-γ promotes the phenotype switch of smooth muscle cells [71] and triggers the secretion of C-X-C motif chemokine ligand 10. This, in turn, could inhibit healing responses in the endothelium, as recently demonstrated in a mouse model of arterial injury [5]. Consequently, targeting proinflammatory T-cells and preventing smooth muscle cell transformation could be beneficial in stabilizing the endothelium and reducing the likelihood of erosion.

## Plaque Erosion Models

Human arteries have a more complex tunica intima than mice [11]. This difference in extracellular matrix composition between species could potentially impact the initiation of the disease. However, mouse models of atherosclerosis can replicate many aspects of human disease by developing complex lesions [20]. Defining the specific processes involved in plaque erosion has been challenging due to the lack of an optimal animal model for testing. The variations in the extracellular matrix between species may be particularly important in plaque erosion, as vascular proteoglycans are abundant at the culprit site. To investigate the impact of T cells in atherosclerotic lesions on plaque erosion, more accurate models that reflect the progression of plaques leading to erosion are necessary. A deeper understanding of the various stages of human atherosclerosis is vital for constructing these models. Despite improvements in animal models, humans themselves will continue to be the best model for studying atherosclerosis [20]. Another proposal found in recent literature, which requires an erosion model for testing, is the notion that matrix metalloproteinase 9 may cleave the functional domain of CD31 on CD4 + T cells [68, 72]. This cleavage could result in the dysregulation of T cells in plaque erosion.

The features of human plaque erosion have been successfully replicated in a mouse model of endothelial denudation [67]. An electrical injury is induced in the left common carotid artery, followed by the application of a constrictive cuff to induce arterial flow perturbation. This leads to neointimal hyperplasia and the accumulation of hyaluronan in a replicative manner [67]. Endothelial detachment exposes the basement membrane containing type IV collagen, resulting in platelet activation. Neutrophils and their extracellular traps can further promote this process, leading to a thrombus rich in platelets, similar to human disease [27]. This model allows for mechanistic studies on the factors contributing to the final steps of the disease. As previously mentioned, in vitro studies have shown that low molecular weight fractions of hyaluronan activate toll-like receptor 2 signaling. In the mouse model, it was demonstrated that toll-like receptor 2 participates in endothelial cell activation, amplifies the recruitment of neutrophils after flow perturbation, and exacerbates erosions. Another recent study using a clonal hematopoiesis model with *Jak2*^V617F^ bone marrow transplanted *Ldlr*^−/−^ mice, showed that it impairs endothelial integrity, increases neutrophil extracellular traps, and worsens superficial erosions. This leads to increased platelet recruitment and enhanced thrombosis [73]. In a different model of wire-induced injury to the left carotid artery, the re-endothelialization process was found to be affected by T cells [5]. The extent to which this model accurately reflects the erosion process is uncertain since no neointimal hyperplasia or hyaluronan accumulation has been described.

## Targeted Drug Delivery to the Erosion Site and Other Treatment Strategies

There is a distinction between plaque erosion and plaque rupture in terms of platelet biology and function [27]. This has led to the hypothesis that medical treatment with dual antiplatelet therapy can be beneficial in treating acute coronary events without the need for percutaneous coronary intervention in selected cases. A small prospective clinical study called EROSION (Effective Anti-Thrombotic Therapy Without Stenting: Intravascular Optical Coherence Tomography-Based Management in Plaque Erosion) tested this hypothesis [74]. The study enrolled 60 patients with acute coronary syndrome attributed to plaque erosion, and antithrombotic therapy was administered without a preceding percutaneous coronary intervention. After one month, 78% of patients had reduced their thrombus by more than 50% according to optical coherence tomography. After one year, medical management was associated with a lower incidence of new plaque layer formation and less plaque progression [75]. Thus, conservative treatment with anti-thrombotic therapy without stenting may be a good option for cases of plaque erosion. Further research is needed to improve noninterventional management and larger trials are needed to test the efficacy and its effect on cardiovascular outcomes.

Treatments that directly target the erosion site are currently being developed. One example is the use of nanoparticles that specifically target plaque rupture and erosion [76]. When exposed to ultrasound, these nanoparticles can undergo a phase change and transform into gas microbubbles. By targeting class A scavenger receptors, these specially designed nanoparticles can be taken up by macrophages in rupture-prone plaques. Upon activation of the nanoparticles, these macrophages undergo apoptosis. The nanoparticles also target eroded plaques by binding to cyclic RGD, which binds to glycoprotein IIb/IIIa on activated platelets and promotes platelet disaggregation [76]. While this approach is innovative, a better target for the underlying cause of plaque erosion would be necessary. Additionally, a more specific approach to resolving plaque inflammation would be helpful. Another type of nanoparticle preparation can target type IV collagen to selectively deliver drugs to areas of denuded endothelium [77]. In a mouse model, protein arginine deiminase-4 inhibitors were delivered to intimal injuries to inhibit the formation of neutrophil extracellular traps and preserve endothelial integrity. This could be significant in the medical management of acute plaque erosions. Another proposed treatment for erosion is the pharmacological inhibition of myeloperoxidase, which helps limit endothelial dysfunction in vascular inflammation [78].

However, we recommend considering preventive measures targeting T-cell-driven tissue inflammation, as it has a significant impact on early lesion development. T-cell-based therapeutics have been proposed for atherosclerosis and are increasingly utilized for various other diseases [79]. A recent example of this is the use of PD1-targeted antibodies in cancer treatment [80]. This treatment has demonstrated the ability to regulate certain T-cell responses in atherosclerotic plaques and slow down disease progression.

## Conclusions

The initial question we posed was whether there is an inherent difference in the atheromatous substrate between plaque erosions and ruptures. Histopathological studies have shown that the plaques have distinct compositions, but further research is needed to understand the morphological transitions that predispose to either manifestation. Our proposal is that T cells play a role in the processes leading up to the superficial erosion of plaques. Aberrant inflammation resolution can result in an erosion plaque phenotype, characterized by excessive matrix generation and weakened endothelial integrity. While there have been reports of direct cytotoxic actions by T cells on the endothelium, the histopathology of the lesions suggests an indirect role for T cells through interaction with smooth muscle cells.

The rapid progress in the field of plaque erosion will continue to enhance our understanding of this pathological process, with potential impact on the management of patients with acute coronary syndrome and other clinical manifestations of atherosclerosis. The identification of specific biomarkers that can be used at the point of care, without the need for invasive imaging, would bring us closer to precision medicine. There is evidence supporting the idea that plaque erosion is a distinct pathological and clinical entity within the broader spectrum of acute coronary syndrome, although the underlying cause remains the same: atherosclerosis. In some respects, plaque erosion could be considered an earlier manifestation than plaque rupture. This suggests that different preventive approaches should be adopted for plaque erosion. However, there is currently a lack of animal models that fully reflect the disease spectrum, highlighting the need to develop reliable models to study the erosion process. The use of optical coherence tomography has revealed subgroups of eroded plaques, but this modality has limitations and better tools are needed to detect vascular inflammation and plaque T cells in situ.

## Key References

Important (●) and very important (●●) references within the past 3 years with brief explanations of their importance. 

- Libby P. Inflammation during the life cycle of the atherosclerotic plaque. Cardiovasc Res. 2021;117(13):2525-36. 10.1093/cvr/cvab303.
  - A state-of-the-art narrative review on the role of inflammation in atherosclerosis, introducing the concepts upon which this review is based.

- Lorenzo C, Delgado P, Busse CE, Sanz-Bravo A, Martos-Folgado I, Bonzon-Kulichenko E, et al. ALDH4A1 is an atherosclerosis auto-antigen targeted by protective antibodies. Nature. 2021;589(7841):287-92. 10.1038/s41586-020-2993-2.
  - A high-throughput single-cell analysis of the antibody repertoire associated with atherosclerosis in mice.

- Gerhardt T, Seppelt C, Abdelwahed YS, Meteva D, Wolfram C, Stapmanns P, et al. Culprit plaque morphology determines inflammatory risk and clinical outcomes in acute coronary syndrome. Eur Heart J. 2023;44(38):3911-25. 10.1093/eurheartj/ehad334.
  - An optical coherence tomography study that distinguishes differences in the inflammatory proteome between plaque rupture and erosion in coronary blood samples.

- Cornelissen A, Gadhoke NV, Ryan K, Hodonsky CJ, Mitchell R, Bihlmeyer NA, et al. Polygenic Risk Score Associates With Atherosclerotic Plaque Characteristics at Autopsy. Arterioscler Thromb Vasc Biol. 2024;44(1):300-13. 10.1161/atvbaha.123.319818.
  - An histopathological study evaluating polygenic risk scores for coronary artery disease in cases of sudden death that identified disparate associations for plaque rupture and erosion.

- Satta S, Beal R, Smith R, Luo X, Ferris GR, Langford-Smith A, et al. A Nrf2-OSGIN1&2-HSP70 axis mediates cigarette smoke-induced endothelial detachment: implications for plaque erosion. Cardiovasc Res. 2023;119(9):1869-82. 10.1093/cvr/cvad022.
  - A mechanistic study that recreated features of endothelial erosion in vitro and both found new therapeutic targets and potential biomarkers for erosion in patients who smoke.

- Chowdhury RR, D’Addabbo J, Huang X, Veizades S, Sasagawa K, Louis DM, et al. Human Coronary Plaque T Cells Are Clonal and Cross-React to Virus and Self. Circ Res. 2022;130(10):1510-30. 10.1161/circresaha.121.320090.
  - A study using single-cell technology and in vitro assays to define the phenotype of activated T cells at different stages of human atherosclerosis as well as their reactivity towards autoantigens produced by smooth muscle and endothelial cells.

- Zhao TX, Sriranjan RS, Tuong ZK, Lu Y, Sage AP, Nus M, et al. Regulatory T-Cell Response to Low-Dose Interleukin-2 in Ischemic Heart Disease. NEJM Evid. 2022;1(1):EVIDoa2100009. 10.1056/EVIDoa2100009.
  - A clinical trial that evaluated the feasibility and safety of targeting adaptive immunity in atherosclerosis using recombinant interleukin-2.

- Kawai K, Sakamoto A, Mokry M, Ghosh SKB, Kawakami R, Xu W, et al. Clonal Proliferation Within Smooth Muscle Cells in Unstable Human Atherosclerotic Lesions. Arterioscler Thromb Vasc Biol. 2023;43(12):2333-47. 10.1161/atvbaha.123.319479.
  - A novel approach to examine clonality of smooth muscle cells in human atherosclerosis.

## Data Availability

No datasets were generated or analysed during the current study.
